# Characterizing advanced breast cancer heterogeneity and treatment resistance through serial biopsies and comprehensive analytics

**DOI:** 10.1038/s41698-021-00165-4

**Published:** 2021-03-26

**Authors:** Allen Li, Jamie M. Keck, Swapnil Parmar, Janice Patterson, Marilyne Labrie, Allison L. Creason, Brett E. Johnson, Molly Downey, George Thomas, Carol Beadling, Laura M. Heiser, Annette Kolodzie, Alexander R. Guimaraes, Christopher L. Corless, Joe W. Gray, Gordon B. Mills, Raymond C. Bergan, Zahi I. Mitri

**Affiliations:** 1 Knight Cancer Institute, Oregon Health and Science University, Portland, OR USA; 2 Center for Spatial Systems Biomedicine (OCSSB),Oregon Health and Science University, Portland, OR USA; 3 Department of Biomedical Engineering, Oregon Health and Science University, Portland, OR USA; 4 Department of Diagnostic Radiology, Oregon Health and Science University, Portland, OR USA; 5 Department of Pathology, Oregon Health and Science University, Portland, OR USA; 6 Department of Cell, Developmental & Cancer Biology, Oregon Health and Science University, Portland, OR USA; 7 Department of Systems Biology, The University of Texas MD Anderson Cancer Center, Houston, TX USA

**Keywords:** Breast cancer, Breast cancer, Cancer genomics, Molecular medicine, Tumour heterogeneity

## Abstract

Molecular heterogeneity in metastatic breast cancer presents multiple clinical challenges in accurately characterizing and treating the disease. Current diagnostic approaches offer limited ability to assess heterogeneity that exists among multiple metastatic lesions throughout the treatment course. We developed a precision oncology platform that combines serial biopsies, multi-omic analysis, longitudinal patient monitoring, and molecular tumor boards, with the goal of improving cancer management through enhanced understanding of the entire cancer ecosystem within each patient. We describe this integrative approach using comprehensive analytics generated from serial-biopsied lesions in a metastatic breast cancer patient. The serial biopsies identified remarkable heterogeneity among metastatic lesions that presented clinically as discordance in receptor status and genomic alterations with mixed treatment response. Based on our study, we highlight clinical scenarios, such as rapid progression or mixed response, that indicate consideration for repeat biopsies to evaluate intermetastatic heterogeneity (IMH), with the objective of refining targeted therapy. We present a framework for understanding the clinical significance of heterogeneity in breast cancer between metastatic lesions utilizing multi-omic analyses of serial biopsies and its implication for effective personalized treatment.

## Introduction

Heterogeneity in breast cancer can be observed across metastatic lesions (intermetastatic heterogeneity [IMH]) within an individual patient and can even be seen within a singular lesion (intratumoral heterogeneity)^1,2^. Heterogeneity is observed at many biological levels, including morphologic, phenotypic, and molecular. Furthermore, both spatial and temporal heterogeneity can affect clinical outcomes in breast cancer^3,4^. Therefore, biopsies of multiple lesions at multiple time points provide a more comprehensive profile of a patient’s cancer and are critical for selecting the most appropriate therapy. Current algorithmic treatment guidelines do not sufficiently address the complexity of IMH and its therapeutic challenges, representing an unmet need in heterogeneous diseases like breast cancer.

Classification of breast cancer based on the clinical immunohistochemistry (IHC) status of estrogen receptor (ER), progesterone receptor (PR), and human epidermal growth factor receptor 2 (HER2) is fundamental in clinical subtyping, prognostication, and treatment selection. Although the receptor status is largely preserved throughout treatment course, there are clinically significant exceptions. Receptor status alterations have been documented following neoadjuvant chemotherapy in loco-regional breast cancer^5–7^, as well as between matched primary and metastatic breast cancer lesions^8–10^. Reports suggest that ER, PR, and HER2 changes following neoadjuvant treatment or throughout tumor progression is a potential indicator of poor prognosis when taken into consideration with the tumor’s original receptor status^6,11–13^. A recent study of primary breast cancers found HER2 discordance following neoadjuvant HER2-directed therapy was associated with a reduced disease-free survival^14^. Receptor status discordance may represent tumor heterogeneity resulting from clonal selection based on therapeutic stress^10,15,16^. Routine ER, PR, and HER2 IHC assays may provide an initial sampling of heterogeneity, but these markers provide a limited picture of tumor biology and may not always correlate with gene expression levels^17^. Beyond the clinical receptor status, gene expression profiling with breast cancer intrinsic subtyping further informs tumor characterization. PAM50 intrinsic subtypes (luminal A, luminal B, HER2-enriched, basal, and normal-like) can reveal changes in RNA expression of *ESR1*, *PGR*, and *ERBB2* along with genes involved in processes such as proliferation and cell cycle^18^. Discordance of intrinsic subtypes between primary and metastatic tumors have been reported^10^. Studies comparing primary and metastatic lesions observed conversion to more aggressive molecular subtypes within luminal and HER2-enriched intrinsic subtypes, often due to decreased luminal-related genes and increases in proliferation and migration genes ^19,20^.

In addition to heterogeneity identified in receptor status, IMH can also manifest as subclonal genetic alterations within critical signaling and growth pathways. Studies comparing metastatic breast cancer to primary tumors have shown that while they share many genomic alterations, metastatic breast cancers can exhibit different genomic profiles than early stage disease, including acquisition of driver alterations not present in the primary disease^21–24^. Metastatic tumors may also have an increased mutational burden and/or higher subclonal diversity compared to primary tumors^21,25^. Phylogenetic analysis of metastatic breast cancer within individual patients has indicated that two modes of disease progression exist, one in which all metastases are monoclonal, sharing a common metastatic origin, and another in which multiple metastases arise from different subclones within the primary tumor^26^, leading to molecularly heterogeneous metastases. IMH can also play a significant role in patient response to treatment and resistance. A recent study found different clinically significant genetic alterations in *PIK3CA* across metastatic sites within the same individual, who had a mixed response to PI3 kinase (PI3K) inhibitor treatment^27^. In addition, *ESR1* mutations arise with aromatase inhibitor (AI) treatment and contribute to resistance to therapy^28,29^. Therefore, the navigation of treatment for heterogeneous tumors may benefit from clinical tumor profiling data provided by repeat biopsies that inform rational and effective precision therapy combinations, taking into account disease-type, toxicities, and emerging resistance mechanisms^30^.

The mechanisms underlying IMH and the evolutionary paths of metastatic lesions in individual patients remain poorly understood. There is no routine clinical assays or reliable predictive models for consistently assessing IMH throughout the disease course and under therapeutic pressure. Because of the effect of IMH on prognosis and treatment resistance, there is a compelling need to develop a reliable means for assessing IMH in clinical settings to enable rational modification or addition of therapy. We have previously described the design of our Serial Measurements of Molecular and Architectural Responses to Therapy (SMMART) program in the context of workflows for successfully acquiring tissue in real-time from patients with advanced cancer and performing comprehensive clinical and exploratory analytics on the specimens^31^. Here we discuss the importance of repeat metastatic biopsies in capturing clinically significant IMH and this program’s multi-omic translational oncology approach in an individual breast cancer patient, with a focus on understanding the role of IMH in treatment response.

## Results

### Clinical description

A 35-year-old female presented with sudden onset of blurry vision in her left eye. She had a history of right breast stage IIb (pT2N1M0) ER/PR positive, HER2-normal (non-amplified), infiltrative ductal carcinoma that was diagnosed four years previously. At her initial presentation, she was treated with bilateral mastectomies and right axillary lymph node dissection, adjuvant anthracycline/taxane based chemotherapy, and endocrine therapy with tamoxifen. Germline genetic testing with the Myriad MyRisk breast panel did not identify pathogenic mutations in the tested genes, including *BRCA1/2*.

Metastatic evaluation including ophthalmologic exam showed metastatic disease involving the left choroid (leading to visual symptoms), pathologic adenopathy (mediastinal, paratracheal, prevascular, suprahilar, and subcarinal), and multiple bilateral pulmonary metastasis. A mediastinal node biopsy was positive for recurrent metastatic breast carcinoma, ER/PR positive and HER2-normal (1+) by IHC. She underwent radiotherapy to the choroid lesion.

**Fig. 1:**
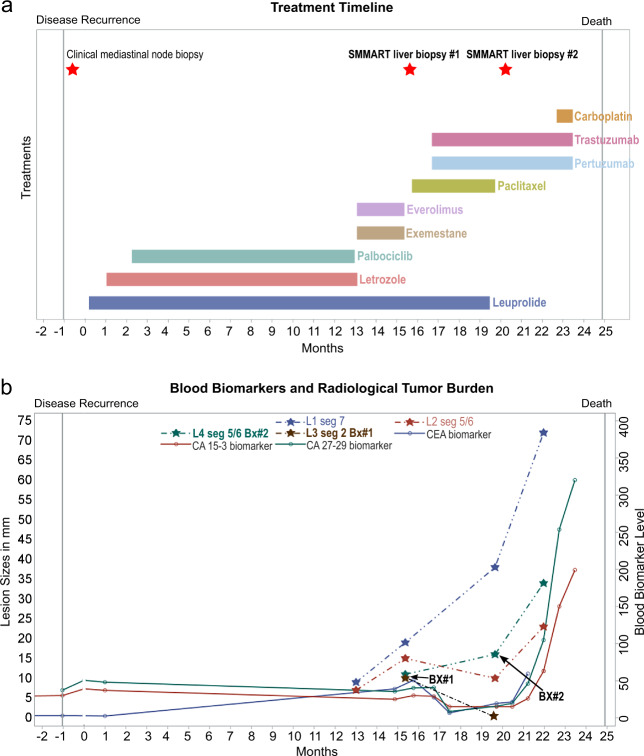
Overview of clinical timeline and response. **a** Treatment (colored boxes) and biopsy (red stars) timeline, in months, following disease recurrence in the metastatic setting. **b** Timeline of blood biomarker levels (U/ml, solid lines) and lesion sizes (in millimeters) for four selected hepatic lesions (hatched lines). Study biopsy #1 (Bx#1) = Liver 3 segment 2 (L3 seg2); Study biopsy #2 (Bx#2) = Liver 4 segment 5/6 (L4 seg5/6).

At this time, the patient was started on systemic therapy with palbociclib and letrozole with ovarian function suppression. Treatment course outlined in Fig. 1a. Despite initial partial response, repeat scans 10 months after treatment initiation showed progression, with multiple new hepatic and osseous lesions. Therapy was changed to everolimus and exemestane, on which she had rapid progression as evident on repeat imaging within 2 months of therapy. A new biopsy was performed under the IRB-approved observational study, “Molecular Mechanisms of Tumor Evolution and Resistance to Therapy” (MM-TERT)^31^, within the precision oncology SMMART-program at Oregon Health and Science University, Knight Cancer Institute. This program aims to benefit individual patients by identifying potential actionable biology through multi-omic analysis of serial metastatic biopsies to guide novel precision combination therapies. Study biopsy #1 of a new lesion, liver 3 segment 2 (L3 seg2 in Fig. 1b) was consistent with metastatic breast adenocarcinoma, ER/PR negative, and HER2 positive (3+) by IHC; different from the prior ER/PR positive and HER2-normal primary and mediastinal lesions. Based on the change in HER2 status, the patient was started on paclitaxel, trastuzumab, and pertuzumab. Remarkably, 3 months after initiation of therapy, restaging scans showed that the Study biopsy #1 lesion (L3 seg2) was resolved (Fig. 1b), along with several other hepatic lesions (not shown in Fig. 1b). However, response was mixed and 12 hepatic lesions did not respond, including L1 seg7 and L4 seg5/6 (Fig. 1b). Given the mixed response, the patient underwent a second biopsy of a progressing lesion, liver 4 segment 5/6 (L4 seg5/6) (Study biopsy #2, Fig. 1b), revealing metastatic breast carcinoma, ER/PR negative, HER2-normal (1+) by IHC, consistent with triple-negative breast cancer (TNBC). New brain metastases were also identified and treated with whole brain radiotherapy. Based on this new data, the patient’s treatment was changed to carboplatin in combination with trastuzumab, and she received two cycles of therapy with improvement in hepatic enzymes. This therapy was complicated by side effects requiring hospitalization. Her performance status deteriorated, and the patient elected to transition to hospice care.

### Multi-omic analytics

To comprehensively characterize tumor biopsy tissue, clinical assays were performed within the OHSU Knight Diagnostic Laboratories that are CLIA-licensed/CAP-accredited, including IHC (ER, PR, HER2, AR, and PD-L1), a targeted next-generation sequencing (NGS) panel covering 125 genes (GeneTrails^©^ Comprehensive Solid Tumor Panel), whole transcriptomic sequencing (Illumina TruSeq RNA exome), and a multiplex protein analysis of 22 key cancer proteins and phosphoproteins (the Intracellular Signaling Protein Panel) developed on the NanoString Vantage 3D^™^ Solid Tumor Panel. In addition, an exploratory assay, reverse phase protein array (RPPA), was used to profile 450 proteins and phosphoproteins. Due to limited material from Study biopsy #1, extended analysis outside of IHC and genomic sequencing could not be performed on this specimen.

### Characterization of heterogeneity in receptor status and intrinsic subtype

**Fig. 2:**
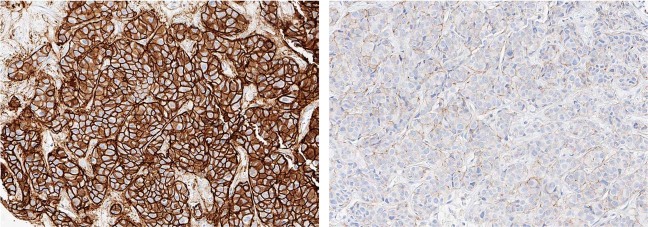
HER2 immunohistochemistry (IHC) comparison of Study biopsies. Strong HER2 IHC staining in the first hepatic biopsy (Study biopsy #1, **left**) and negative HER2 IHC staining in the second hepatic biopsy (Study biopsy #2, **right**). Images are shown at ×20 magnification.

**Fig. 3:**
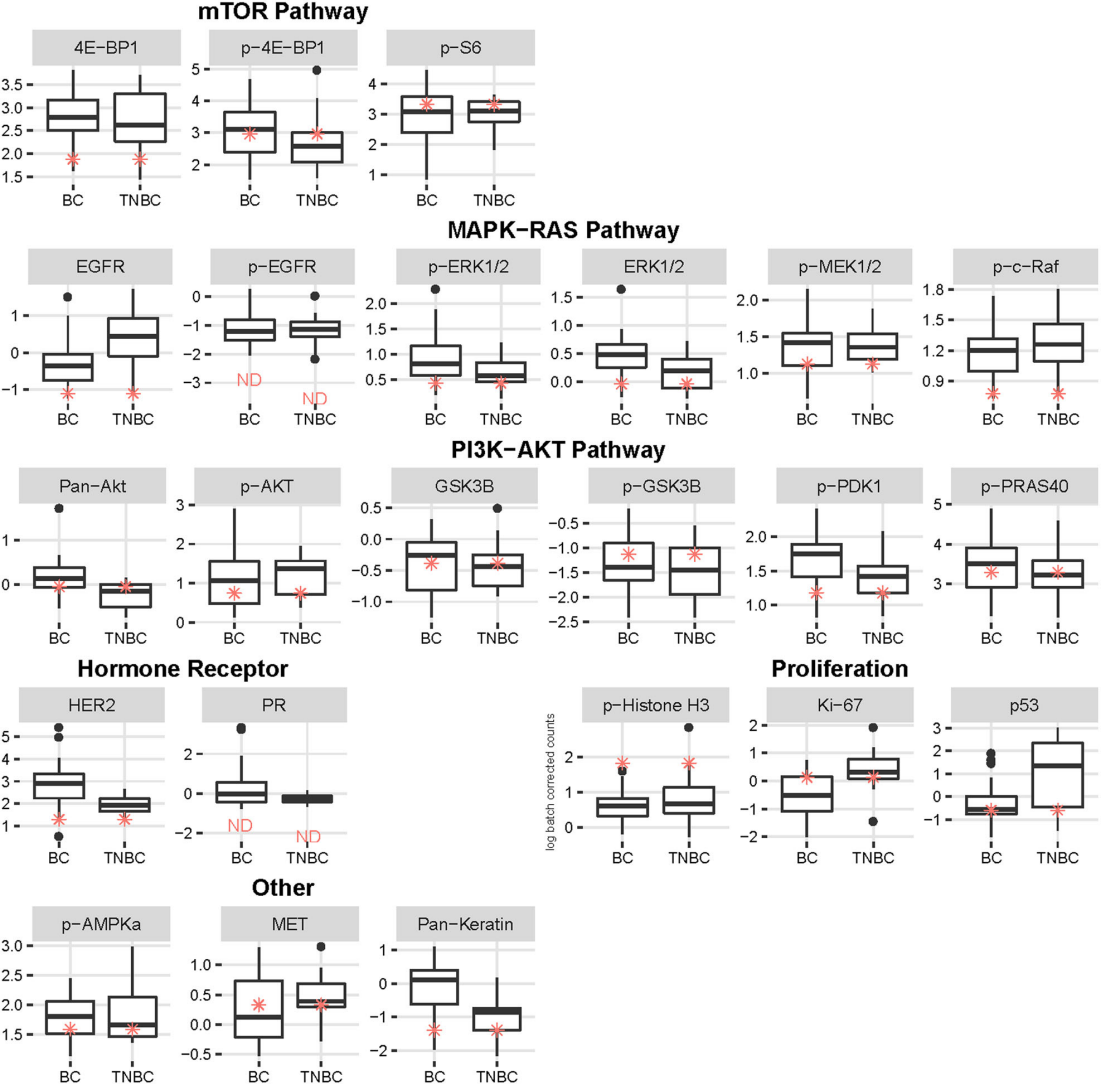
Pathway analysis of protein and phosphoproteins within Study biopsy #2 by the Intracellular Signaling Protein Panel. Box and Whisker plots *Y*-axis showing the distribution of antibody levels (log batch correct counts) of Study biopsy #2 (red asterisks) compared to a cohort of 32 metastatic breast cancers of all subtypes (BC) or 15 metastatic TNBC specimens, *X*-axis. Cohort consists of predominantly HER2 negative samples. Boxes show the 25th, 50th (median line), and 75th percentiles of the cohorts. Protein and phosphoproteins are grouped under pathways including “PI3K-AKT”, “mTOR”, and “MAPK-RAS”, and the categories “Proliferation” and “Other”. ND = Not detectable or below level of background, measured with non-specific antibody.

Examination of receptor status of the study biopsies confirmed the dramatic changes in ER and HER2 levels from the previous breast and mediastinal biopsies. IHC on Study biopsy #1 showed loss of ER expression and gain of HER2 expression: ER negative (0%), PR negative (0%) and HER2 positive (3+, with 80% of tumor cells positive). This HER2 positivity correlated with marked amplification of *ERBB2* (45 copies, Table 1), as detected by NGS. After 4 months of paclitaxel, trastuzumab, and pertuzumab, a progressing hepatic mass (Study biopsy #2) was found to be consistent with TNBC: ER negative (<1%), PR negative (0%), and HER2-normal (1+) by IHC. The tumor was also positive for androgen receptor (AR, 30%). Previous biopsies were not tested for AR; therefore, it is unknown if this was a new hormonal activation. The striking differences in HER2 IHC staining between Study biopsies #1 and #2 are illustrated in Fig. 2. NGS on Study biopsy #2 confirmed that *ERBB2* was not amplified (3 copies, Table 1). Finally, the Intracellular Signaling Protein Panel found very low HER2 protein, when comparing relative level of Study biopsy #2-derived protein expression compared to two cohorts of metastatic breast cancers: all subtypes (BC) and TNBC (Fig. 3, HER2 box plot under “Receptor”).

**Table 1:** Comparison of alterations in Study biopsies identified by NGS panel sequencing.

Genomic alterations	Liver Study Biopsy #1 (5.3 yrs from diagnosis) Following everolimus/exemestane	Liver Study Biopsy #2 (5.7 yrs from diagnosis) Following paclitaxel; On treatment with trastuzumab/pertuzumab
Microsatellite status	MSI Stable	MSI Stable
Gene fusions	Negative	Negative
PIK3CA	p.E542K (29% VAF)	p.E542K (25% VAF)
TP53	Splice site c.375 + 1 G > T (84% VAF)	Splice site c.375 + 1 G > T (74% VAF)
ERRB2	Copy Gain (45 copies)	Not reported (3.2 copies)
CDKN2A	Copy Loss (0 copies)	Not reported (0.7 copies)
MYC	Copy Gain (16 copies)	Not reported (5 copies)
BRCA2	Regional Chr13 Loss (0.16 copies)	Not reported (1.2 copies)
RB1	Regional Chr13 Loss (0 copies)	Not reported (0.7 copies)
CCND1	Regional Chr11 Gain (48 copies)	Regional Chr11 Gain (17 copies)
FGF4	Regional Chr11 Gain (22 copies)	Regional Chr11 Gain (10 copies)
FGF3	Regional Chr11 Gain (18 copies)	Regional Chr11 Gain (9 copies)
FGF19	Regional Chr11 Gain (17 copies)	Regional Chr11 Gain (7 copies)
RICTOR	p.V1358M (30% VAF)	Not reported
CDK12	Copy Gain (10 copies)	Not reported (2 copies)
MTOR	Not reported	p.E706K (8.6% VAF)

Genomic alterations identified in the two study liver biopsies (Study biopsy #1 and Study biopsy #2) taken 4 months apart, highlighting genomic heterogeneity. Not reported = below threshold for reporting and not on clinical report.

*VAF* variant allele frequency.

**Fig. 4:**
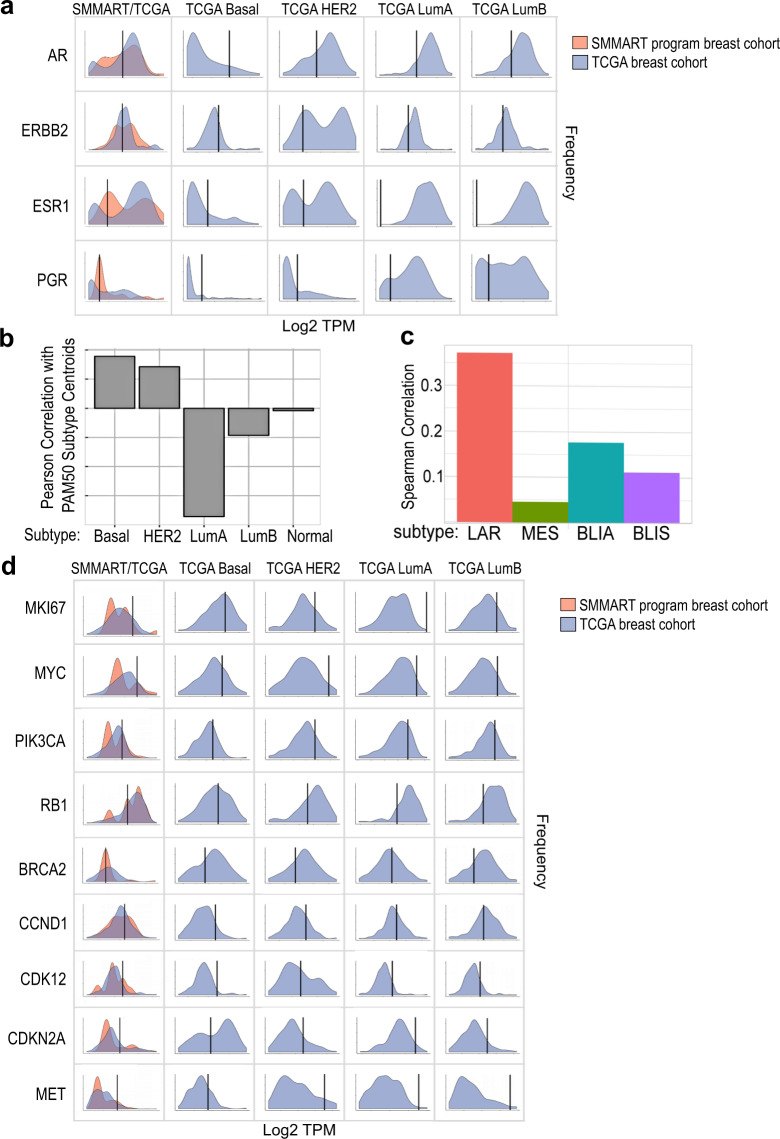
RNA profiling analysis of Study biopsy #2 showing intrinsic subtyping and gene expression of hormone receptors and identified copy alterations. **a** Hormone receptor gene expression: distribution of batch corrected gene expression values, *z*-scaled within each cohort, filtered by subtype, and depicted as a density histogram. *Y*-axis shows the frequency, *X*-axis represents the log transformed transcripts per million (TPM), mean centered and scaled within each cohort: TCGA Breast Cancer cohort (*n* = 1227), in blue, and a SMMART-program metastatic breast cancer cohort (*n* = 40), in orange. Black line marks the patient’s RNA expression for *ERBB2* (HER2), *ESR1* (ER), *PGR* (PR), and *AR*. **b** PAM50 intrinsic subtyping analysis of RNA expression related to five breast cancer subtypes: Basal, HER2, Luminal A (LumA), Luminal B (LumB), and Normal. *Y*-axis is the Spearman Correlation. **c** TNBC-specific intrinsic subtyping: analysis of RNA expression that identifies four basal subtypes: basal-like immune-activated (BLIA), basal-like immunosuppressed (BLIS), mesenchymal (MES) and luminal androgen receptor (LAR). *Y*-axis is the Spearman Correlation. **d** Additional RNA expression profiles of genes found to be altered by genomic or proteomic assays, processed and displayed as in (**a**).

Comparative RNA expression analysis of genes associated with receptor status in Study biopsy #2: *ESR1* (ER), *PGR* (PR), *ERBB2* (HER2) and *AR*, showed high RNA expression in relation to basal intrinsic subtypes from The Cancer Genome Atlas (TCGA) primary breast cancer cohort (Fig. 4a). When compared to other TCGA intrinsic subtype cohorts (HER2, luminal A, and luminal B) and a SMMART-program cohort (40 metastatic breast cancers), *AR* was moderate, while *ESR1* was low. *ERBB2* expression in Study biopsy #2 was moderate compared to luminal A/B TCGA and SMMART-program cohorts. The fact that this immunohistochemical HER2-negative (1+), *ERBB2* non-amplified lesion had moderate *ERBB2* RNA expression could indicate heterogeneity or differential regulation.

PAM50 intrinsic subtyping allows for tumor characterization beyond receptor status, using expression of hormone, cell proliferation, differentiation, and cytokeratin genes^32^. Study biopsy #2 showed a predominantly basal-like intrinsic subtype (Fig. 4b), which was consistent with the clinical receptor status subtype (ER, PR, and HER2 negative). In addition, Study biopsy #2 showed a correlation, albeit lower, to the HER2-enriched intrinsic PAM50 subtype. Analysis of the specific genes within the PAM50 signature that were shared between the basal and HER2-enriched subtypes provided clarification for the detection of both subtypes. Study biopsy #2’s low expression of *ESR1* and *SLC39A6*, combined with moderate to high expression of most proliferation-related genes (such as *MKI67* [Fig. 4d], *CCNB1, CENPF, CDC20, CCNE1, CDC6, and TYMS*) contributed heavily to both the basal and HER2-enriched intrinsic subtypes. High MYC expression was unique to the basal subtype (Fig. 4d). The HER2-enriched subtype in Study biopsy #2 was influenced by high expression of *GRB7* (same amplicon as *ERBB2*), moderate expression of *ERBB2, TMEM45B, FGFR4*, and *FOXA1* and low expression of *MIA, GPR160, SFRP1*. This data reveals an atypical TNBC lesion with characteristics of both basal and HER2 intrinsic subtypes, which may help to inform the treatment strategy. Further characterization of the basal PAM50 subtype with gene expression related to immune, microenvironment, and *AR* provides critical insight into treatment response and survival within TNBC patient population^17,33^. This expanded intrinsic subtyping distinguishes four subtypes: basal-like immune-activated (BLIA), basal-like immunosuppressed (BLIS), luminal androgen receptor (LAR), and mesenchymal (MES)^17^. Study biopsy #2 was LAR (Fig. 4c), which was supportive of AR IHC positivity. Overall, the expression pattern in Study biopsy #2 was most consistent with a basal intrinsic subtype but was not fully aligned with one subtype due to its unique expression within disparate pathways including MYC, HER2, proliferation, and AR.

**Fig. 5:**
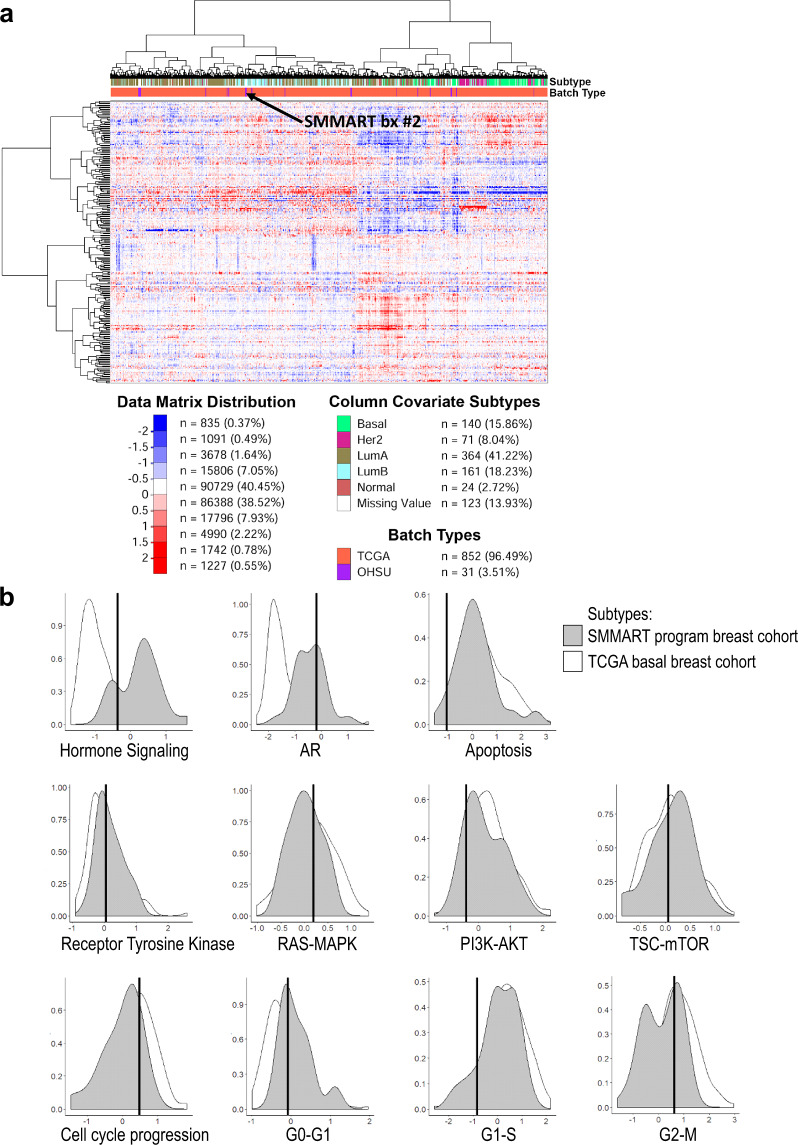
Reverse Phase Protein Array (RPPA) profiling by subtype clustering and pathway analysis of proteins and phosphoproteins in Study biopsy #2. Protein expression values were normalized within the TCGA breast cancer cohort. **a** The heat map represents a rank sum ordering of the protein expression across TCGA (red), 31 SMMART-program samples (purple), and Study biopsy #2 (arrow). The red and blue colors represent higher and lower expression proteins, respectively. **b** Pathway profiling by RPPA. The data were *z*-scored and pathway activity was assessed using pathway scores calculated as described previously^56^. The histogram represents the distribution of the pathway’s activity (*Y*-axis = density) of the TCGA basal breast cancer cohort (white) and SMMART-program cohort (gray) as well as the pathway activity of Study biopsy #2 (black line). See Supplementary Table 1 for proteins comprising each RPPA pathway.

Protein and phosphoprotein levels within Study biopsy #2 were evaluated by RPPA in relation to breast cancer intrinsic subtype clustering (Fig. 5a). The tumor’s protein expression was normalized within the TCGA breast cohort (“batch-types”, red) and a SMMART-program cohort (31 metastatic breast cancers, “batch-types”, blue). Study biopsy #2 did not clearly cluster with any specific intrinsic subtype but primarily clustered within luminal-like subtypes (“subtype”, blue and brown), rather than basal (“subtype”, green). This luminal clustering most likely reflects consequences of AR expression, which was high by RPPA. In contrast, RPPA pathway profiling assessment of the hormone receptor signaling pathway downstream ER signaling was low compared to the SMMART-program cohort but higher than the TCGA basal cohort (Fig. 5b), similar to the analysis of *ESR1* RNA expression (Fig. 4a).

### IMH within cellular proliferation and signaling pathways

Unexpected intermetastatic genomic heterogeneity within cellular proliferation and cell signaling pathways was found by sequencing both Study biopsy #1 (liver lesion with 65% tumor content) and #2 (liver lesion with 75% tumor content) with the GeneTrails^©^ Comprehensive Solid Tumor Panel (Table 1). Study biopsy #1 and #2 shared only two alterations, a *PIK3CA* E542K hot spot mutation (29% and 25% variant allele frequency [VAF], respectively) and a *TP53* splice site mutation (84% and 74% VAF, respectively). In Study biopsy #1, a variant of unknown significance (VUS) within the mTORC2 complex, *RICTOR* V1358M (30% VAF) was identified, while in Study biopsy #2 a VUS within the mTORC1/2 complex was reported, *MTOR* E706K (8% VAF). Copy number alterations in Study biopsy #1 included bi-allelic copy losses in *CDKN2A* (0 copies, p16 negative IHC confirmed), *RB1* (0 copies, RB1 negative IHC confirmed), and *BRCA2* (0.16 copies), and large copy gains in *CCND1* (cyclin D1, 48 copies), *FGF3/FGF4/FGF19* (18/22/17 copies, respectively), *MYC* (16 copies), *CDK12* (10 copies), and *ERBB2* (45 copies). However, Study biopsy #2, obtained ~4 months later, was largely absent of copy number alterations. *CDK12* (2 copies), *ERBB2* (3 copies), and *BRCA2* (2 copies) were wildtype, and a lower level of copy gain in *CCND1* (17 copies), *FGF3/FGF4/FGF18* (9/10/7 copies), and *MYC* (5 copies) was found. Furthermore, only one copy of *RB1* and *CDKN2A* was lost (0.8 and 0.7 copies, respectively).

RNA expression profiling of genes related to G1 cell cycle confirmed a lack of significant copy gains or losses, showing an average gene expression in Study biopsy #2 compared to the SMMART-program and TCGA cohorts (Fig. 4d). However, expression of *MET* and *PIK3CA*, which harbored the hot spot mutation, was high compared to all cohorts. As previously discussed, Ki-67 (*MKI67*) had higher expression compared to all cohorts but that of basal.

Protein profiling by the Intracellular Signaling Protein Panel and RPPA upstream and downstream of PI3K-AKT and MAPK pathways demonstrated little activation within Study biopsy #2. Upstream analysis of receptor tyrosine kinases (RTK) by the Intracellular Signaling Protein Panel showed overall low HER2 (Fig. 3, HER2 box plot under “Receptors”) but above average protein levels for MET in comparison to the BC cohort (MET box plot under ‘Other’). This correlated with results from RPPA pathway profiling that showed an average signal from the RTK pathway (which includes phosphorylated MET and HER2) compared to the basal TCGA and SMMART-program cohorts (Fig. 5b). Downstream signaling was not observed within the MAPK pathway using the Intracellular Signaling Protein Panel, represented by low phospho-cRAF, phospho-MEK, and phospho-ERK1/2 (Fig. 3, box plots under “MAPK-RAS Pathway”). However, RPPA profiling of the MAPK pathway showed an average signal (Fig. 5b). Downstream signaling within the PI3K-AKT pathway was observed, but lower than expected based on genomic evidence of the *PIK3CA* mutation and high expression. Several phosphoproteins within the PI3K-AKT pathway analyzed by the Intracellular Signaling Protein Panel were within the low to average quartiles of the BC and TNBC cohorts, such as phospho-AKT, phospho-PDK1, and phospho-PRAS40, while phospho-GSK3β was above average in both cohorts (Fig. 3, box plots under “PI3K-AKT Pathway”). Within the mTOR pathway, phospho-S6 and phospho-4E-BP1 were within the higher quartile of the TNBC cohort (Fig. 3, box plots under “mTOR Pathway”). RPPA profiling of the PI3K pathway was comparatively low, while the TSC-mTOR pathway was average (Fig. 5b). Feedback from the mTOR pathway could contribute to the apparent low PI3K-AKT activity. Minor discrepancies in pathway results from the two assays may be explained by the larger number of proteins defining the RPPA pathway profiles (see RPPA Supplementary Table 1), the analysis of different biopsy cores, and differences in comparator cohorts.

Protein profiling of cell cycle and proliferation demonstrated a high mitotic index. Phosphorylated histone-H3, a mitotic marker on the Intracellular Signaling Protein Panel, was high compared to both cohorts, whereas interphase cell proliferation assessed by Ki67 was moderate compared to the BC cohort and lower than the highly proliferative TNBC cohort (Fig. 3, box plots under “Proliferation”). RPPA pathway profiling revealed a high cell cycle progression signal, which includes markers from all cell cycle phases (Fig. 5b). Deeper analysis of cell cycle phases from individual pathway plots, showed that this was not due to cyclin D1 (G0-G1) or S phase cell cycle regulators (G1-S), but high signal from G2/M that may reflect slow progression through the later cell cycle phases. Furthermore, RPPA identified low apoptotic signal, consistent with the tumor cells being resistant to apoptosis.

## Discussion

Identification and assessment of IMH in real-time is an urgent challenge, as it is becoming increasingly established that heterogeneity can affect cancer treatment selection and prognosis. Breast cancer receptor discordance, as signified by a change in ER, PR, or HER2 receptor status, represents IMH that is critical in treatment selection and correlates with poor outcome. IMH presenting as receptor discordance has been documented between primary and metastatic lesions in breast cancer. Multiple studies have indicated that receptor status can be dynamic and may evolve during the course of treatment and tumor progression^8,13,34^. Studies showed an overall 16–30% receptor status change after neoadjuvant treatments^35,36^, with change in HER2 status being less common than that of ER or PR^35–37^. Between primary and metastatic breast cancer, a meta-analysis of 39 studies showed a total discordance rate of 10.3% for HER2, 19.3% for ER, and 30.9% for PR^9^. Similar findings were confirmed by two other recent reports^38,39^, showing HER2 discordance rate of 8.5% and 10%. Most recently, a study with matched primary and metastatic tumors showed 24.6% and 36.9% discordance based on receptor status and PAM50, respectively^10^. However, receptor discordance among different metastatic lesions through tumor progression remains poorly understood, likely due to the lack of routine serial biopsies across metastatic sites. Our study directly addresses this important knowledge gap. It demonstrates that the patient’s metastatic lesions were discordant among biopsies, starting as ER/PR positive, HER2-normal in both primary and initial mediastinal metastasis, then ER/PR negative, HER2-positive in the liver Study biopsy #1, and finally TNBC (with AR positivity) in the liver Study biopsy #2.

Given the clinical and therapeutic implications of IMH, it is important to perform biopsies on metastatic lesions under specific circumstances in breast cancer to capture the heterogeneity in order to aid treatment selection. The first hepatic Study biopsy #1 was performed due to the unexpected rate of disease progression through two lines of treatment prescribed for a presumed ER/PR positive, HER2-normal breast cancer based on a prior biopsy. Specifically, the patient had rapid progression on both palbociclib and everolimus-based therapies. The MONALEESA-7 study showed that median progression free survival (PFS) for premenopausal women with ER/PR positive, HER2-normal, advanced breast cancer receiving similar cyclin-dependent kinase 4/6 (CDK4/6) inhibitor therapy is ~23 months, much longer than the 10 months seen in this case^40^. While *CCND1* amplification and *CDKN2A* loss have been associated with sensitivity to CDK4/6 inhibition in some studies^41,42^, the co-occurring loss of *RB1* found in the biopsy is associated with resistance^43,44^ and its likely presence in additional lesions may be responsible for the shorter PFS. After progression on palbociclib and letrozole, the patient received everolimus and exemestane, which in the second line setting has PFS ranging from 8.5 to 11 months^45–47^, again, significantly better than the 2 months for this patient. On progression, repeat imaging identified new lesions including both study biopsy lesions (L3 seg2 and L4 seg5/6, Fig. 1b) that were resistant to everolimus. Due to the limited specimen from Study biopsy #1, we were unable to explore mechanisms of resistance to everolimus outside of *ERBB2* amplification, such as a potential lack of downstream mTOR signaling or activation of compensatory pathways. Overall, a biopsy of the progressing metastatic lesion (Study biopsy #1) was pursued given the unusual clinical course and uncovered a HER2-positive lesion, allowing for treatment change accordingly.

Additional metastatic biopsies are warranted when treatment response is suboptimal, compared to expected results, in order to inform treatment decisions. Molecular profiling can provide insights into resistance mechanisms or further characterization of the tumor. The first imaging event following paclitaxel, trastuzumab, and pertuzumab treatment showed complete resolution of five lesions, including the Study biopsy #1 lesion that harbored the *ERBB2* amplification; however, the patient had overall mixed response to HER2-directed therapy. Study biopsy #2 was performed on a liver lesion that dramatically progressed on trastuzumab and pertuzumab alone in order to understand this lesion’s biology and identify potentially targetable alterations. Clinical characterization revealed a TNBC tumor, with low HER2 protein level that was corroborated by the Intracellular Signaling Protein Panel. Intrinsic RNA subtyping showed a basal PAM50 subtype (Fig. 4b) based on its high proliferation-related gene expression and MYC but also indicated moderate expression of *ERBB2* RNA. Additional TNBC-specific intrinsic subtyping showed an LAR subtype (Fig. 4c) suggesting luminal attributes due to *AR* expression, and RPPA supported this heterogeneous phenotype (Fig. 5a). The clinical data along with some insight from exploratory assays revealed mechanisms of resistance to HER2-directed therapy and prior endocrine therapy (everolimus and AI combination). *AR* expression and resulting luminal characteristics can contribute to AI resistance and can decrease efficacy of chemotherapy and everolimus combinations in TNBC tumors, even with PI3K pathway mutations ^48,49^.

Study biopsy #2’s genomic landscape was quite different from the targeted alterations found in the previous biopsy, taken only 4 months prior. *PIK3CA* and *TP53* mutations were still present, but surprisingly five of six clinically relevant copy number alterations were absent, including those in cell cycle regulators and *ERBB2*. This genomic profile was corroborated by RNA and protein profiling of the lesion showing little to no expression changes or deregulation within G1 or S cell cycle phases or cell signaling pathways. The exception being high G2/M and phospho-histone-H3 signal, with downregulated apoptosis. The repeat metastatic biopsy offered clinical implications in the patient’s treatment and provided biological insight into this heterogeneous cancer in terms of genomic alterations and adaptive RNA and proteomic regulation. The variable clinical response of the four hepatic lesions tracked in the clinical timeline (Fig. 1b) suggests the possibility of further heterogeneity among metastatic lesions that were not biopsied. Ideally, repeat biopsies of multiple lesions at the same time would help fully assess the temporal and spatial nature of IMH and its evolution. Unfortunately, this can lead to increased procedural risk and undue burden for the patient, which needs to be balanced carefully.

The origin and mechanisms leading to IMH remain under investigation. Multiple driving forces likely contribute to IMH, including subclonal expansion from selective treatment pressure, tumor microenvironment composition and function, cellular plasticity, and genomic instability^1,16,50^. The dramatic differences in *ERBB2* and other copy alterations between the biopsied lesions could have resulted from selective pressure for pre-existing ER-negative subclonal populations that arose, with subsequent branching of clones with or without *ERBB2* amplification. This would result in IMH causing heterogeneous response to HER2 treatment and would benefit from strategic combination treatments for effective tumor control. HER2-directed treatment can result in tumor evolution and the loss of HER2 overexpression in some lesions^51^, which could be possible even with only 4 months of treatment. HER2 treatment resistance can also result from high RNA *MET* expression, as was observed in Study biopsy #2 (Fig. 4d). HER2-positive basal-like breast cancers can activate MET with expression of its ligand, the hepatocyte growth factor (HGF), particularly in liver where HGF is high. For HER2 lesions with a known resistance mechanism, such as *MET* expression, preclinical studies have shown that adding crizotinib to the HER2 treatment in effort to reconstitute HER2 dependency is encouraging and should be explored further^52^. IMH beyond receptor status can be explored with tumor genome sequencing. The alterations identified in this case are generally consistent with our current understanding of the metastatic breast cancer genomic landscape, with *PIK3CA* and *TP53* mutations and *CCND1* copy alterations being the most common^21–23^. Previous studies showed genomic alterations between primary and matched metastatic lesions in ER positive breast cancer are largely concordant^25^, which highlights the unusual molecular divergence between the two metastatic lesions in this patient. The possibility that the different metastatic lesions originated from separate primary lesions cannot be excluded. In clinical practice, a different or occult primary tumor can very-well contribute to IMH and should not be overlooked. However, while not conclusive, we believe these two metastatic lesions are derived from the same progenitor neoplastic cell population based on the presence of the same *PIK3CA* E542K and *TP53* splice site mutation in both samples identified by NGS. Both mutations were determined to be somatic and the likelihood that they arose independently in the same individual is low. Furthermore, low pass whole genome sequencing to assess chromosomal copy alterations in both biopsies showed an overall similar chromosomal gain and loss profile, providing further evidence that these subclonal lesions originated from the same primary clone (data not shown). Rather, our findings could be consistent with a recent phylogenetic study that identified copy number alteration differences between the primary and metastatic lesions, as well as among metastatic lesions^26^. This study suggested that independent seeding events from a primary tumor can give rise to genetically distinct metastatic lesions and that genomic divergence can be found at various time points, including after the establishment of metastatic disease^26^. A process that could lead to a population of diverse subclones in neoplastic disease is telomere crisis during transformation. Early in breast cancer, a critical transition and crisis event occurs, where shortened telomeres are rescued by re-expression of telomerase, allowing for immortalization and widespread genome instability in ductal carcinoma in situ as cells transition to invasive cancer^18^. This tumor could have undergone this early event, as evidence of chromosome instability and high levels of phosphorylated histone-H3 suggests delays in mitosis that could be due to defects in chromosome division. This was supported by the RPPA cell cycle signatures, specifically finding G2/M phases to be the most affected. The exact mechanism and source behind the receptor discordance throughout this patient’s course remained elusive. However, despite its source, capturing this discordance during her metastatic treatment course allowed informed therapy refinement.

While the mechanisms that give rise to tumor heterogeneity remain incompletely understood, managing tumor heterogeneity clinically remains a challenge, especially in the cases of heterogeneous lesions harboring mutations that suggest different treatment sensitivities. In current practice, repeat clinical biopsies are rarely performed in the metastatic setting, which in our experience might result in missed opportunities in identifying clinically significant IMH. While routine serial biopsies may not be indicated or feasible, clinical scenarios of unanticipated rapid progression or mixed response should prompt suspicion of clinically significant IMH and consideration for a repeat biopsy to refine therapy options. Evaluation of IMH is possible using a serial approach that prioritizes the most aggressive lesion for biopsy, which may change throughout the clinical course. Measuring blood-based serum biomarker levels can assist with monitoring and provide a more frequent, non-invasive assessment of treatment response that can prompt an imaging or biopsy event. In addition, liquid biopsies based on sequencing of cell free DNA from peripheral blood has emerged as a promising technique to monitor genetic alterations from many different lesions, and should be explored in future studies to closely monitor arising heterogeneity and treatment response. With the development of new targeted agents in breast cancer and wider implementation of multi-omic analyses, analytical assessment of IMH holds investigational promises. The paths to implementation of and financial coverage for the emerging assays are not yet clear and need to be established. It is important to note that the multi-omic translational oncology approach remains investigational and clinical tumor profiling should remain focused on receptor status and actionable mutations with approved matched therapies. However, serial biopsies with appropriate testing should be encouraged in selected clinical circumstances as presented above. Further study will be necessary to better understand the temporal and spatial nature of IMH and to better predict and target changes under therapeutic pressure.

## Methods

### Patient enrollment, tissue processing, and DNA extraction

Specimens and data were acquired from the participant by obtaining informed written consent to use their coded de-identified data and/or specimens for research and publication purpose under regulation by the Oregon Health & Science University (OHSU) IRB# 16113 MM-TERT. Formalin-fixed paraffin-embedded biopsies and tumor tissue were collected and DNA extraction was carried out using a FFPE DNA extraction kit (QIAGEN). DNA was extracted from plasma and buffy coat using Macherey-Nagel NucleoSnap and QIAgen Blood and Tissue kits, respectively. DNA isolated from both FFPE samples and buffy coat were fragmented by sonication to 150 bp using a Covaris E220 prior to library preparation.

### Clinical immunohistochemistry

Tissue is formalin-fixed for ~12 h (meets ASCO/CAP guidelines). Immunohistochemical stains are performed on formalin-fixed, paraffin-embedded tissue, using a biotin-free protocol (Ventana Ultraview) that includes appropriate positive and negative controls. All antibodies are sourced through Ventana and come pre-diluted. The ER (Catalog# 790-4324, Clone: SP1, Rabbit monoclonal, 1 µg/mL), PR (Catalog# 790-2223, Clone: 1E2, Rabbit monoclonal, 1 µg/mL), and Her-2/neu (Catalog# 790-2991, Clone: 4B5, Rabbit monoclonal 6 µg/mL primary antibody) immunohistochemical stains are performed with FDA status 510(k) cleared kits from Ventana according to manufacturer’s instructions, with appropriate controls. OHSU participates in proficiency testing for ER, PR and Her-2/neu IHC.

### NGS: GeneTrails^®^ comprehensive solid tumor panel

Preparation of DNA and sequencing was performed at the Knight Diagnostics Laboratories. Total nucleic acid was extracted from macrodissected, tumor-rich areas from FFPE sections was purified and used for NGS. 50 ng of DNA was used to prepare a custom amplicon-based sequencing library (QiaSeq) that covers target exons and flanking intronic sequences for 125 cancer-associated genes. 100 ng of RNA was converted to cDNA and amplified using another custom QiaSeq library that can detect gene fusions involving 24 clinically informative target genes in a partner-agnostic approach. Sequencing was performed on an Illumina NextSeq500.

### RNA transcriptome sequencing

Preparation of RNA and transcriptome sequencing was performed at the Knight Diagnostics Laboratories using the TruSeq RNA Access library preparation kit and sequenced on the Illumina NextSeq500. Approximately 100 million reads were generated per sample. RNA sequence reads were processed following the methods described by Tatlow & Piccolo^53^. Briefly, sequenced reads were trimmed with Trim Galore (http://www.bioinformatics.babraham.ac.uk/projects/trim_galore/) using default parameters. Trimmed reads were quantified for transcript expression by Kallisto to the GENCODE release 24 reference transcriptome (https://pachterlab.github.io/kallisto)^54^.

### RNA expression profiling

Expression abundance in transcripts per million (TPM) were batch corrected using batch control replicates with removal unwanted variation method. Expression levels were compared by mean centered and scaled gene expression within the cohort of 40 metastatic breast cancers SMMART-program samples to the mean centered and scaled expression levels of TCGA breast cohort.

### PAM50 intrinsic subtyping/TNBC-specific intrinsic subtyping

PAM50 analysis of 50 genes that correlate with five breast cancer subtypes (basal, normal, HER2, luminal A and luminal B), and TNBC-tumor subtype analysis of 77 genes that correlate with four TNBC subtypes (BLIA, BLIS, LAR, and MES). The sample is assigned to a molecular subtype with the highest Spearman correlation between the subtype’s centroid and the corresponding gene expression pattern^32,55^.

### Intracellular signaling protein panel

This clinical assay is based on the Vantage 3D Protein Solid Tumor Panel (FFPE) available from Nanostring Technologies. It consists of a cocktail of 24 oligonucleotide-tagged antibodies designed to bind to specific proteins involved in intracellular signaling or to specific sites of phosphorylation on these proteins. In addition, non-specific mouse IgG1 and rabbit IgG antibodies are included as controls for background binding (total of 26 antibodies). The oligonucleotides are joined to the antibodies via a UV-sensitive chemical linker. Following heat-induced epitope retrieval in citrate buffer, 5 micron sections of tumor core biopsies and control FFPE cancer cell lines were incubated with the antibody cocktail for 12 h at 4 degrees. After washing, slides were placed on a UV light box for 3 min, and released oligonucleotides were collected, hybridized to code sets, and counted on a Nanostring MAX nCounter system. Signals from the FFPE cell lines were used to normalize the data from different runs.

### Reverse phase protein array

Protein extracts from tumor samples were analyzed with a panel of 450 proteins and phosphoproteins as previously described^56,57^. In order to scale the protein expression values, the RPPA data from the patient sample was merged within the TCGA primary breast cancer and SMMART-program metastatic breast cancer RPPA datasets, using the replicate-based normalization method^58^. The protein expression values were then *z*-scored by using the median and standard deviation and a heat-map was generated from the Study biopsy #2. The heat map was produced using publicly available Cluster 3.0 and TreeView software.

### Protein pathway analysis

RPPA Pathways were calculated as previously described^57^. Proteins used as predictors of the different pathways are listed in Supplementary Table 1. To determine a pathway score, for each sample, all positively associated predictors were summed minus the predictors that are negatively associated with the pathway. The total was then divided by the numbers of predictors in the pathway. To generate the pathway scores histograms, the distribution of TCGA basal subtype breast cancer samples and 31 SMMART metastatic breast cancer samples were plotted and the value of the patient sample was added to the histograms.

### Reporting summary

Further information on research design is available in the Nature Research Reporting Summary linked to this article.

## Supplementary information

Supplementary Information

Reporting Summary

## Data Availability

The raw RNA sequencing data generated during the current study, are available in the dbGaP repository: https://identifiers.org/dbgap:phs002321.v1.p1^59^. As these files are controlled access, researchers must request access to the dbGaP data. The repository also includes clinical and phenotypic metadata and molecular data (including gene and protein expression). The normalized gene expression (RNAseq) data and the reverse phase protein array data (protein expression data), are publicly available in the Synapse repository under the following project accession: syn22975916^60^. HER2 immunohistochemistry data, Intracellular Signaling Protein Panel assay data, and data from the GeneTrails Solid Tumor Panel assay, are not publicly available, but will be made available on reasonable request. Please contact the Knight Diagnostic Laboratories at Oregon Health and Science University (OHSU), email: KDLClientServices@ohsu.edu, for more information on these datasets. The TCGA RNAseq and the TCGA RPPA data analyzed during the study, are available in the Open Science Framework repository: https://osf.io/gqrz9/^61^. The data generated and analyzed during this study are described in the following metadata record: 10.6084/m9.figshare.13615712^62^.
