# Supplementary material for: Characterizing advanced breast cancer heterogeneity and treatment resistance through serial biopsies and comprehensive analytics
Source: NPJ Precis Oncol. 2021 Mar 26;5:28. doi: 10.1038/s41698-021-00165-4 (PMC7997873; doi:10.1038/s41698-021-00165-4)
Supplement: Supplementary file 1 — Supplementary Information [file 41698_2021_165_MOESM1_ESM.pdf]

Supplemental Table 1: RPPA pathway proteins

Pathway	Predictor	Weight
Hormone_receptor	AR	1
Hormone_signaling	BCL2	1
	ERALPHAPS118	1
	GATA3	1
	INPP4B	1
Apoptosis	Caspase-7 -cleaved	1
	Caspase-8 -cleaved	1
Cell_cycle_progression	14-3-3-beta	-1
	CyclinD1	-1
	p21	-1
	p27_pT198	-1
	cdc25C	1
	CDK1_pT14	1
	Chk1	1
	Cyclin-B1	1
	PLK1	1
	Rb_pS807_S811	1
G0_G1	BRD4	-1
	Cyclin-B1	-1
	14-3-3-beta	1
	53BP1	1
	Cyclin-D1	1
	p21	1
	p27_pT198	1
G1_S	53BP1	1
	BRD4	1
	Cyclin-E1	1
G2_M	cdc25C	1
	CDK1_pT14	1
	Cyclin-B1	1
	PLK1	1
	Rb_pS807_S811	1

Pathway	Predictor	Weight
PI3K_Akt	INPP4b	-1
	PTEN	-1
	Akt_pS473	0.5
	Akt_pT308	0.5
	GSK-3a-b_pS21_S9	1
	p27_pT198	1
	PRAS40_pT246	1
	Tuberin_pT1462	1
RAS_MAPK	B-Raf_pS445	1
	c-Jun_pS73	1
	C-Raf_pS338	1
	JNK_pT183_Y185	1
	MAPK_pT202-Y204	1
	MEK1_p_S217-S221	1
	p38_pT180_Y182	1
	p38-MAPK	1
	p90RSK_pT573	1
	YB1_pS102	1
RTK	Src_pY416	0.5
	Src_pY527	0.5
	CMET_pY1235	1
	EGFR_pY1173	1
	HER2_pY1248	1
	HER3_pY1289	1
	IGF1R_pY1135_Y1136	1
	IRS1	1
	Shc_pY317	1
	SHP-2_pY542	1
TSC_mTOR	S6_pS235_S236	0.5
	S6_pS240_S244	0.5
	4E-BP1_pS65	1
	mTOR_pS2448	1
	p70-S6K_pT389	1
	Rb_pS807_S811	1
	Rictor_pT1135	1
