# Supplementary material for: Characterizing advanced breast cancer heterogeneity and treatment resistance through serial biopsies and comprehensive analytics
Source: NPJ Precis Oncol. 2021 Mar 26;5:28. doi: 10.1038/s41698-021-00165-4 (PMC7997873; doi:10.1038/s41698-021-00165-4)
Supplement: Supplementary file 2 — Reporting Summary [file 41698_2021_165_MOESM2_ESM.pdf]

nature research

Corresponding author(s): Zahi Mitri

Last updated by author(s): Feb 10, 2020

Reporting Summary

Nature Research wishes to improve the reproducibility of the work that we publish. This form provides structure for consistency and transparency in reporting. For further information on Nature Research policies, see our Editorial Policies and the Editorial Policy Checklist.

Statistics

For all statistical analyses, confirm that the following items are present in the figure legend, table legend, main text, or Methods section.

n/a	Confirmed	
☒	☐	The exact sample size (*n*) for each experimental group/condition, given as a discrete number and unit of measurement
☒	☐	A statement on whether measurements were taken from distinct samples or whether the same sample was measured repeatedly
☒	☐	The statistical test(s) used AND whether they are one- or two-sided<br>*Only common tests should be described solely by name; describe more complex techniques in the Methods section.*
☒	☐	A description of all covariates tested
☒	☐	A description of any assumptions or corrections, such as tests of normality and adjustment for multiple comparisons
☒	☐	A full description of the statistical parameters including central tendency (e.g. means) or other basic estimates (e.g. regression coefficient) AND variation (e.g. standard deviation) or associated estimates of uncertainty (e.g. confidence intervals)
☒	☐	For null hypothesis testing, the test statistic (e.g. *F*, *t*, *r*) with confidence intervals, effect sizes, degrees of freedom and *P* value noted<br>*Give P values as exact values whenever suitable.*
☒	☐	For Bayesian analysis, information on the choice of priors and Markov chain Monte Carlo settings
☒	☐	For hierarchical and complex designs, identification of the appropriate level for tests and full reporting of outcomes
☒	☐	Estimates of effect sizes (e.g. Cohen's *d*, Pearson's *r*), indicating how they were calculated

Our web collection on statistics for biologists contains articles on many of the points above.

Software and code

Policy information about availability of computer code

Data collection	No custom software used. Patient data collected from EPIC electronic medical record. Clinical assays: IHC, GeneTrails Solid Tumor Panel, RNA Transcriptome, and Intracellular Signaling Protein Panel were run by the Knight Diagnostic Laboratories (https://knightdxlabs.ohsu.edu/home/research-services/overview). RPPA assay was ran at the M.D. Anderson core (https://www.mdanderson.org/research/research-resources/core-facilities/functional-proteomics-rppa-core.html).
Data analysis	Information available in methods section:<br>RNA sequence reads were processed with Trim Galore (http://www.bioinformatics.babraham.ac.uk/projects/trim_galore/) using default parameters. Trimmed reads were quantified for transcript expression by Kallisto to the GENCODE release 24 reference transcriptome (ref 53: Bray NL et al., Near-optimal probabilistic RNA-seq quantification. Nat Biotechnol. 2016;34(5):525-7.)<br><br>Intrinsic subtyping: The sample is assigned to a molecular subtype with the highest Spearman correlation between the subtype's centroid and the corresponding gene expression pattern (refs: 31, 54; Parker JS, et al. Supervised risk predictor of breast cancer based on intrinsic subtypes. J Clin Oncol. 2009;27(8):1160-7 and Wallden B, et al. Development and verification of the PAM50-based Prosigna breast cancer gene signature assay. BMC Med Genomics. 2015;8:54.)<br><br>RPPA: RPPA data from the patient sample was merged within the TCGA primary breast cancer and SMMART-program metastatic breast cancer RPPA datasets, using the replicate-based normalization method (ref 57: Akbani R, et al. A pan-cancer proteomic perspective on The Cancer Genome Atlas. Nat Commun. 2014;5:3887.) RPPA heat map was produced using publicly available Cluster 3.0 and TreeView software. RPPA Pathways were calculated as previously described (ref 56: Labrie M, et al. Adaptive responses in a PARP inhibitor window of opportunity trial illustrate limited functional interlesional heterogeneity and potential combination therapy options. Oncotarget. 2019;10(37):3533-46.).

For manuscripts utilizing custom algorithms or software that are central to the research but not yet described in published literature, software must be made available to editors and reviewers. We strongly encourage code deposition in a community repository (e.g. GitHub). See the Nature Research guidelines for submitting code & software for further information.

Data

Policy information about availability of data

All manuscripts must include a data availability statement. This statement should provide the following information, where applicable:

- Accession codes, unique identifiers, or web links for publicly available datasets
- A list of figures that have associated raw data
- A description of any restrictions on data availability

The raw RNA sequencing data generated during the current study, are available in the dbGaP repository: https://identifiers.org/dbgap:phs002321.v1.p1 (1). As these files are controlled access, researchers must request access to the dbGaP data. The repository also includes clinical and phenotypic metadata and molecular data (including gene and protein expression). The normalized gene expression (RNAseq) data and the reverse phase protein array data (protein expression data), are publicly available in the Synapse repository under the following project accession: syn22975916 (2). HER2 immunohistochemistry data, Intracellular Signaling Protein Panel assay data, and data from the GeneTrails Solid Tumor Panel assay, are not publicly available, but will be made available on reasonable request. Please contact the Knight Diagnostic Laboratories at Oregon Health and Science University (OHSU), email: KDLClientServices@ohsu.edu, for more information on these datasets. The TCGA RNAseq and the TCGA RPPA data analyzed during the study, are available in the Open Science Framework repository: https://osf.io/gqrz9/ (3). The data generated and analysed during this study are described in the following metadata record: https://doi.org/10.6084/m9.figshare.13615712 (4).

REF

1. . dbGaP https://identifiers.org/dbgap:phs002321.v1.p1. Updated 2021.

2. Li A, Keck JM, Parmar S, Patterson J, Labrie M, et al. Characterizing Advanced Breast Cancer Heterogeneity and Treatment Resistance Through Serial Biopsies and Comprehensive Analytics. Synapse https://www.synapse.org/#!Synapse:syn22975916/wiki/606342.

3. Tatlow PJ. Google Cloud Pilot RNA-Sequencing for CCLE and TCGA. Open Science Framework https://osf.io/gqrz9/.

4. Li A, Keck JM, Parmar S, Patterson J, Labrie M, et al. Metadata supporting the article: Characterizing Advanced Breast Cancer Heterogeneity and Treatment Resistance through Serial Biopsies and Comprehensive. figshare https://doi.org/10.6084/m9.figshare.13615712.

Field-specific reporting

Please select the one below that is the best fit for your research. If you are not sure, read the appropriate sections before making your selection.

☒ Life sciences ☐ Behavioural & social sciences ☐ Ecological, evolutionary & environmental sciences

For a reference copy of the document with all sections, see nature.com/documents/nr-reporting-summary-flat.pdf

Life sciences study design

All studies must disclose on these points even when the disclosure is negative.

Sample size	N/A, case report
Data exclusions	N/A
Replication	N/A, single patient case report
Randomization	N/A, single patient case report
Blinding	N/A, single patient case report

Reporting for specific materials, systems and methods

We require information from authors about some types of materials, experimental systems and methods used in many studies. Here, indicate whether each material, system or method listed is relevant to your study. If you are not sure if a list item applies to your research, read the appropriate section before selecting a response.

Materials & experimental systems

n/a	Involved in the study	
☒	☐	Antibodies
☒	☐	Eukaryotic cell lines
☒	☐	Palaeontology and archaeology
☒	☐	Animals and other organisms
☐	☒	Human research participants
☐	☒	Clinical data
☒	☐	Dual use research of concern

Methods

n/a	Involved in the study	
☒	☐	ChIP-seq
☒	☐	Flow cytometry
☒	☐	MRI-based neuroimaging

Human research participants

Policy information about studies involving human research participants

Population characteristics	Single patient case report
Recruitment	N/A
Ethics oversight	Patient consented to observation / tissue collection IRB approved protocol

Note that full information on the approval of the study protocol must also be provided in the manuscript.

Clinical data

Policy information about clinical studies

All manuscripts should comply with the ICMJE guidelines for publication of clinical research and a completed CONSORT checklist must be included with all submissions.

Clinical trial registration	N/A, observation protocol
Study protocol	IRB #16113, Oregon Health and Science University Institutional Review Baord
Data collection	EPIC electronic medical record - Date of consent till date of death
Outcomes	N/A
